# Large Relics Scenario-Based Visualization Using Head-Mounted Displays

**DOI:** 10.1155/2021/2813819

**Published:** 2021-10-05

**Authors:** Hui Xu, Jiawan Zhang

**Affiliations:** ^1^School of Computer Software, College of Intelligence and Computing, Tianjin University, Tianjin, Zip Code 300072, China; ^2^Henan Institute of Science and Technology, Xinxiang, Henan, Zip Code 453000, China

## Abstract

Most visitors come to visit museums; in reality, few immersive solutions support the senses experience. Virtual reality (VR) technology attaches the virtual information from the real environment. Applying the VR technology in the 3D relic information display and visualization in the museum field is a hot research issue. However, most current solutions of relics are one-sided, only focusing on the virtual exhibition, lack of associations with actual function, and senses experience, especially the large artistic cultural relics. The scenario-based virtual exhibition solution is an available approach to allow visitors to imitate ancient artist and provide relatively experience in the form of content and sense organ of ancient art. It converts large relics into “digital large relics” and enables experiencing performance of ancient civilization in person. The solution presents relics to the visitors in a more direct and vivid manner and with innovative forms, strong interaction, and intelligence, thereby improving the interests and satisfaction among visitors in this type of relic exhibition. Besides, it also provides visitors with a convenient way to experience and learn ritual and culture. Evaluation and conclusion can be drawn that most participants appreciated this solution in clear interface and completion aspects.

## 1. Introduction

The history museum is considered as a privileged means for communication and provide a physical means to connect us to past cultures [1]. Museum's education role in the cultural fabric of society emanates via the collections' contribution to scholarship. Collections play a central role in making culture accessible to the mass audience [2], and they contribute towards public cultural enlightenment [3]. How to popularize these cultural treasures to public has attracted more and more researchers' attentions [4–6]. However, in these cases, there is only visual sense communication mode with cultural objects for visitors; other communication modes are extremely limited.

To make it possible for visitors to gain more display information about cultural relics and related historical and cultural knowledge when visiting museums, a new concept of digital exhibition has been put forward, that is, the “expanded spatial display” in the physical space of the museum [7]. The concept integrates virtual display into the physical exhibition space of the museum and makes use of the virtual reality (VR) to enhance the visitors' experience of viewing the physical collections.

The VR technology utilizes the 3D graphics generation technology, multisensor interaction technology, and high-resolution display technology to generate a 3D lifelike virtual environment. The VR system, which contains abundant perceptual information and models, generates the same feedback information as in the real world with the help of the 3D interaction technology and complex sensor devices, such as the digital helmets and gamepads, to provide people with the same feeling as in the real world. Characterized by immersion, interaction, and imaginative, the VR system emphasizes the leading role played by people, which enables the information processing system to satisfy the demands of people and match the senses of people.

With more attention focused on Chinese traditional culture, finding the suitable approach to display the relic information and transmit the historical knowledge to visitors are becoming more necessary. 3D virtual exhibition expands the museum's display space and provides a full range of information about the exhibits, which is a great opportunity for developing the display methods of physical museums. As one of the representatives of large cultural relics, chime bells [8] (or called bianzhong, an ancient Chinese musical instrument) have been studied by some Chinese research teams at the digital experience of chime and its ritual and musical culture.

**Figure 1:**
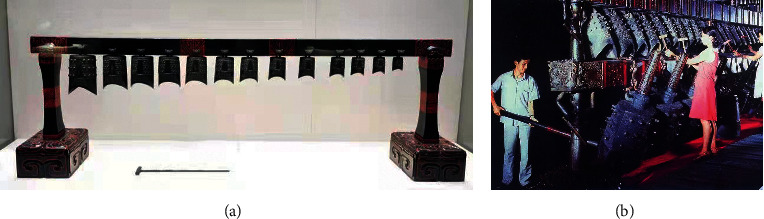
The chime bells unearthed in China. (a) The first chime bells unearthed in China: a unitary ensemble of 13 bells made in Chu State of the Warring States Period collected in the National Museum of China; the largest bell is 30.5 centimeters high and the smallest bell is 13 centimeters high. (b) The scenes of playing the chimes on the replica of Marquis Yi of Zeng chimes: during the playing, the middle- and upper-layer chimes are hit with T-shaped mallets, while the lower-layer large Yong bells are hit with long round wooden sticks [10].

Yang et al. first used the digital technology in the virtual interactive design of chime ritual and musical culture [9]. They used the 3D computer graphics technology to reconstruct the 3D models of chimes, players, and buildings. Through action capturing technology, they collected movement information from the players. Then, they synthesized chime music and used the VRML technology to achieve the virtual scene roaming for chime playing. In the 2010 Shanghai Expo, the Hubei Pavilion used multitouch technology to scale down the images of chimes owned by Marquis Yi of Zeng chimes (Figure 1(b)) onto a mirror glass. Viewers can tap the chimes with hands. The sounds are actual sounds recorded by the Hubei Provincial Museum and are controlled by shadow recognition. Later, the Hubei Provincial Museum used 3D printing technology to restore high-precision cultural relic models of the Marquis Yi of Zeng chimes and other seven musical instruments possessed by the museum. It recorded the sounds of the cultural relic restorations and some original pieces. This museum has successively tried touch-screen-based multitouch technology and laser holographic imaging technology to provide visitors with interactive chime playing experience. However, chime bells are a large percussion instrument. Playing them on a small touch screen is very different from playing in both visual effect and playing method, leading to poor visitor experience. For virtual chime bells that adopt laser holographic projection technique, experiential chime bells are significantly different from real chime bells in the way of playing, though it can imitate the form of a real chime bells. These two methods cannot meet the requirements of visitors and music learners for experiencing and learning music theories and techniques of chime bells.

These technologies satisfied the desires of visitors to interactively play the chimes, but the playing was different from the real way and the experience was poor. There are three reasons:

- Chime percussion requires special hit tools (Figure 1)
- The acoustic characteristics of “two-tone bell” cannot be simulated
- The sensory experience is not real enough, especially the haptic experience

Immersive VR is probably one of the most appealing and potentially effective technologies to solve this problem. The immersive display and visualization can also change the traditional visiting form from only seeing relics and listening to the expositor to a new form that visitors can see the real relic, listen to the explanation, and observe and operate the virtual information of the relic.

This paper construct the “expanded spatial display” for the chime based on the VR technology; the contributions of this paper are as follows:

1. Compared with the desktop VR on the mobile phone screen, the head-mounted displays method based on spatial location can sense spatial movement. The VR exhibition method proposed in this paper simulates the performance of real musical instruments. The digital information content spatially displayed is expanded to provide visitors with personalized browsing and interaction modules. The VR technology changes the traditional way users visit museums, and the users will directly interact with cultural relics, which fills the trip with interaction and exploration.
2. With the VR presentation mode, the characteristic cultural information of large musical instruments can be designed with a variety of digital information, providing a software and hardware platform for researches on the rites and music culture conveyed by the chime, so as to make the display design more technological, intelligent, and diversified. Visitors can compare the information in the real space with that in the virtual space, thereby strengthening the visitors' interest in the exhibits.
3. UX metrics constructed by the proposed study show that, based on measurement of sensory and cognitive in virtual environment, increasing the amount of feedback to a user improves the immersion and thus the overall experience for a user. It has better comprehensive statistic information, better perception performance measurement, and better human factors metrics.

## 2. Related Work

As digital information technology progresses, there emerges a new mode to communicate with digitalized relic in museum and culture heritage fields. The key to approach the general public is the use of new technologies and novel interaction paradigms. With the emergence of digital museum, visitors' communication with historical relic exhibits becomes proactive. People can take the initiative to learn about digitized relic exhibits or conduct more active exchange of information with cultural relics in e-museum, digital exhibition room, or intelligent exhibition hall [11], for example, establishing an e-museum by using the Internet or setting up digital multimedia exhibition halls within traditional museums by using VR, augmented reality, mixed reality, and Web technologies [12–15]. Various researches have been explored in the immersive environment. Firstly, the comparison between data in VR and 2D data visualization has been conducted. Millais et al. [16] concluded that users feel more satisfied and successful when using VR data exploration tools. Petridis et al. pointed out that modern exhibitions have evolved from static exhibition to dynamic exhibition and challenging exploration, and they introduced two applications developed for improving immersion, attraction, and interactivity of user experience in Herbert Art Gallery and Museum [17]. Thomas et al. pointed out that the rapid progress of technology and constantly increasing user needs to pose a new challenge for the 21st-century museums [18]. They introduced that in 2009, Natural History Museum of Britain launched an augmented reality system named Attenborough Studio in London, and the museum utilized augmented reality technology to make a 45-minute interactive movie with this device. Walczak et al. have developed the Augmented Representation of Cultural Objects (ARCO) system [19]. The ARCO system allows museum curators to build, manage, archive, and present virtual museum exhibitions based on the integration of 3D models of artifacts accompanied by images, text, metadata information, sounds, and movies. They allow the public to engage in the immersive experience in the virtual environment, which greatly enhances their satisfaction with the visiting experience. In terms of experience evaluation, Sylaiou et al. discussed the relationship between presence and enjoyment especially for ARCO system [20]. Madsen and Madsen [21] proposed a hand-held visual representation system of castle chapel ruin and needed a large screen TV to display the immersive content and hand-held tablets to present the 3D visualization. Also, studies conducted by some scholars mainly focus on digital museum users. For example, scholar Marty investigated the role played by digital museum in audience's life and pointed out that the audience hopes to see the exhibition closer to actual results, so digital museum should also provide visitors with the unique experience that they cannot experience in a physical museum [22].

## 3. Scenario-Based Chime Bells Application

### 3.1. System Goal and Functions

System goal analysis is an important part in application research. The visualization system developed in this paper addressed three major design goals:

  (G.1) Displaying basic text descriptions of cultural relics: the information functions of digital cultural relics mainly include text introduction and historical allusions.
  (G.2) Giving the user a basic understanding of the structural function analysis of Chime bells: displaying immersive virtual models of cultural relics and allowing visitors operate these models through various user interactions. Visitors observe the 3D relic, rapidly and firstly realizing the exterior design, structure, and material of the current relic.
  (G.3) Giving the user a basic understanding of how the researches for realistic musical Instruments performance experience processes occur: VR-chime bells have the consistent performance function as the real instrument. There are feelings of beating chime bells while applying feedback, auditory feedback, and visual feedback to experiencers and experience of simulating how gavel knock chime bells play and acoustical principle of “a two-tone bell”(people can hear two different tones by striking different part of the bell, and this is called the two-tone acoustic characteristics) of chime bells.

### 3.2. Design and Implementation of Virtual Instruments

Virtual cultural relic chime experience system is a combination of VR and music archeology. The subject took the first chime bells (Figure 1(a)) to be unearthed in China, which are now collected in National Museum of China, as a sample. Besides restoring full-scale bronze chime bells of Chu State in the Warring States Period visually, the virtual bronze chime bells allow experiencers to experience relatively real chime bells playing both in form and in content.

The design of this subject in the VR environment uses HTC VIVE handle as the hit tool to hit the chimes for playing. Based on the acoustic theories of chimes, combining the immersive, interactive, and conceptual features of VR technology, we made some attempts on the chime music playing rules and experiencing methods in virtual environments. It is a novel VR application system and a combination of software and hardware design.

#### 3.2.1. Architecture of Hardware

**Figure 2:**
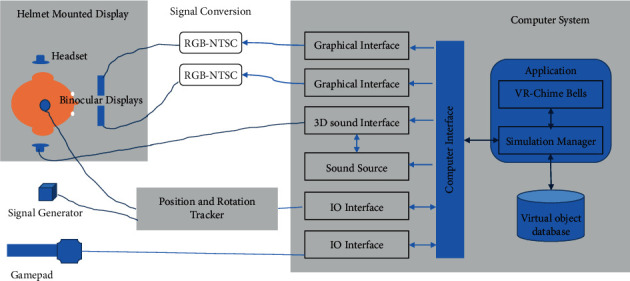
Hardware support system.

As shown in Figure 2, according to the input device, it can be divided into headset interaction and gamepad handle interaction. All these interactions need hardware support.

HTC VIVE allows users to move within a certain space, which is the chime virtual playing space. The VR development platform invokes and controls the two handheld controllers to complete the events of the virtual chimes and commands from human, as well as the interaction and feedback between human and chimes in the VR environment.

The positioning system tracks the monitor and two handle controllers within this space. The immersive application uses the Lighthouse optical positioning framework. Such accurate optical tracking technology can offer better experience, which has advantages, such as small amount of required computing resources, unapparent delay, and a large number of simultaneously tracked objects.

#### 3.2.2. Architecture of the System

**Figure 3:**
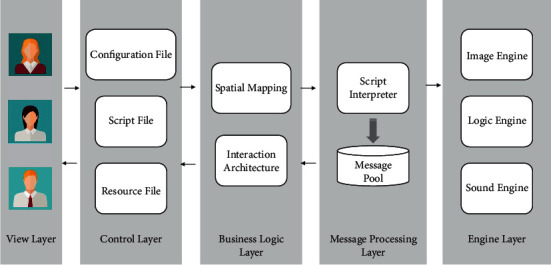
Architecture of the system.

Figure 3 plots the design architecture of the chime exhibition system based on the VR technology; a detailed description of each of these layers is as follows:

- The engine layer includes image engine, logic engine, sound engine, and support for the development of VR applets.
- The message processing layer consists of the script interpreter and message pool. If users input and output instructions in the system, corresponding messages will be released. Both script and instruction messages will enter the message pool, and the message processor will call each module in the engine to process these messages.
- The business logic layer includes system space mapping and digital object interaction, which constitutes the important business logic in the system.
- The control layer mainly receives requests from users, invokes the corresponding business processing object to execute the business module according to different requests, obtains the execution results, and returns appropriate view components to users based on the current state data and the processing results of the business logic.
- The view layer accepts the users' requests and forward these requests to the control layer.

#### 3.2.3. Task

Boring defined the object of human-computer interaction as behavior, namely, people's behavior created and supported by media role of products; he pointed out creating and supporting human activities through the mediating influence of products [23]. Its properties were no longer the physical properties, such as function, structure, and material and the like on which traditional interaction design usually focused when the behavior process (operation process) was the design object of the human-computer interface system. Interaction results from “action” and “feedback” of the system. During system design, user action analysis is the subject of human-computer interface process design. Corresponding tasks are designed according to user behavior. Based on tasks in the goal analysis, visitor behaviors in this application can be divided into three parts: conceptual relic information guidance, presentation of the internal structure, and the properties of the instrument shown correctly.

In terms of conceptual guidance, the Info Detail View displays the detailed text description and animation. The Info View and Info Detail View show the basic text information of relics, and a 360-degree display of virtual chimes to make users enter into an unconscious state, completing the G.1 in the goal analysis.

This system enables users to obtain knowledge of cultural relics by directly clicking on digital cultural relics. By dividing the structure of the digital cultural relic model, this system adds interactive commands for each component so that users can “disassemble” the cultural relics to learn about the detailed information of each component. In this way, users can directly interact with digital cultural relics, thereby completing G.2 in goal analysis.

To enhance user experience, G.3 should be completed. Visitors can play the chime bells to experience the acoustic principle of “two-tone bell” and get the haptic feedback during the hitting process. This design enables the visitors to experience real chime bells playing in both the form and content and also provides a convenient way to experience and learn chime ritual and musical culture for museum visitors, music learners, and cultural heritage professionals. Besides, the method is also an exploration on how to experience and learn Chinese ancient music art in virtual environments and how to develop the art as a kind of digital entertainment.

### 3.3. Scenario-Based Method of Virtual Relics Visualization

The system is comprised of three primary components: (1) user interface (UI) and relevant events, which include the initial interface, help interface, and all UI elements in system; (2) virtual scene system, which used 3DS Max and Unity3D to create the virtual model and Virtual Multisensory Immersion Environment of the virtual chime bells; visualization renders the effect of real-time interaction in a virtual scene to the screen via the HTC VIVE head-mounted display; (3) interactive control, where the function goal of an operational control is to be able to meet the requirements of interactive visualization, including two aspects: it enables a user to directly select and accurately manipulate a model in real time, and it analyzes the incoming message through the virtual interaction scenario control interface and then gives the corresponding interactive control instruction to implement the performance of virtual relic chime and observe the structure in a multidimensional viewpoint. It will provide immersive 3D visualization of chime bells and an overall good user experience.

#### 3.3.1. Application Interface

The scene-based virtual chime system ultimately serves users, so the interactive advantages of VR are highlighted during the design process. The interactive design follows the basic principles of being easy to understand and operate. The design is simplified as much as possible, and the most natural reactions of most users are regarded as an important reference for the design.

First of all, the majority of users are completely unfamiliar with VIVE devices. During operation, users may encounter two kinds of confusion: one is they do not know how to operate a certain function; the other is they do not know the existence of some functions. So, the system provides some simple and efficient operational guidance to encourage users to actively explore and learn, and the pavilion uses functional guidance to let experiencers quickly know what a virtual chime pavilion is, what functions it has, how to achieve these functions, and the specific steps. Before the users use this system, the operators should briefly teach the users so that they can rapidly adapt to the system. To save the teaching cost, it is more convenient to add animations showing users how to use the system. Therefore, a guidance interface of this VR system is designed for users to help them master the experiencing system faster and feel more pleasure from the experience. It can tell the users what this system is, what functions it has, and how to use it. When users first approach the virtual chime pavilion, it needs to quickly convey the concept of the system to them and make them get a good impression and more knowledge about the system so that the system can present itself fully when users use it.

The user-centered interactive design considers the actual needs and using habits of users of all ages to the greatest extent. To help users conveniently gain knowledge about cultural relics, the system presents users with visualized knowledge of cultural relics in the following two ways:

1. Graphical user interface is such an interactive design that places the clicking button near the digital cultural relics in the form of a spatial exhibition board. Users can check information about the cultural relics by clicking the button event in the user interface. The user gazes at the button event in the user interface through the cursor to perform the click behavior, and after that, knowledge about the digital cultural relics will be introduced. Users can perform personalized operations on the digital cultural relics based on their interests and hobbies, thereby gaining insight into related knowledge of cultural relics.
2. Natural user interface is an interactive design that enables users to directly manipulate digital cultural relics. By directly clicking on the digital cultural relics to obtain relevant knowledge, users can learn about each component of the cultural relics in detail, which is similar to the effect of disambiguation and explaining. To make users more familiar with the system, cursor feedback is added to each structural component, for example, “highlight the material.” When users move the gamepad to a specific component, the material of that part will be highlighted as a reminder telling users about the interactive design.

#### 3.3.2. Virtual Multisensory Immersion Environment

Human beings sense the environment through visual, auditory, olfactory, haptic, and gustatory senses. The mixture of these can influence our sense about the environment and provides a richer feeling than the feeling when each one of those senses is independently experienced. Compared with traditional VR, immersive VR brings high-fidelity multisensory virtual experiences. As an instrument of cultural relics, a chime involves three senses: sight, hearing, and touch in its virtualization process. In designing the virtual chime playing environment, we leveraged stimulation of visual, auditory, and haptic senses to make the playing experience more natural and realistic.

**Figure 4:**
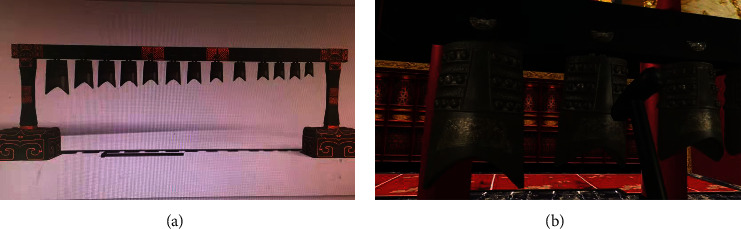
Virtual chime and immersion environment.

*Visual Element Simulation*. The virtual chime pavilion has a virtual exhibition hall similar to an ancient environment. As shown in Figure 4(a), the chimes are also of the same proportion as the original ones and are shown by high-fidelity 3D models. The main body of the visual experience is that human beings can feel the material, texture, and other elements of the bronze chimes in the virtual environment. Meanwhile, the events of hitting the chimes with virtual mallets are mechanically simulated (Figure 4(b)). The swinging of chimes after being hit is natural and real. The swinging amplitudes of different-sized chimes are different. Thus, the visual feedback is accurate and the satisfaction on the playing experience is enhanced.

*Acoustic Simulation of the Two-Tone Bronze Chimes*. The chime bell body is of an “involute-tile shape,” [24] namely, two tiles are involute, with arcs protruding outward. With such a chime body, ancient musicians can get two sounds with different pitches by hitting its central bulge part and side bulge part, respectively [25]. This project simulates the acoustic performance of the two-sound bronze chimes. In the production process, the central bulge part and the side bulge part of each chime are added with two sets of different colliders. Each set of colliders is bound with corresponding music components. The wooden hammer hitting different parts produces different musical scales. Hence, the “two-tone bell” acoustic characteristics can be simulated. The two sounds of a chime body are in a major third relationship, which is in line with the actual acoustic principle of the chimes.

*Haptic Sense Simulation*. The word “haptic” is derived from the Greek “haptesthai,” whose meaning is haptic sense or haptic sense induced. Webster points out that contact sensing is the contacts with the environment felt by the high-bandwidth (50 Hz) sensors near the skin, including the senses about the geometry of a fine surface, the temperature of a wrinkled surface, and sliding [26]. Meanwhile, force feedback is sensed by the low-bandwidth sensors of the human tendon. Although contact sensing and force feedback have some differences in physiology, control requirements, and functions, the sensors can provide the total contact force, the geometry, flexibility, and texture of the grabbed object. The playing tool of the chimes is mallet. The haptic feedback from hitting it is an indispensable sense. Therefore, the subject invokes the HTC VIVE haptic interface and set different vibration frequencies based on the chime sizes to simulate the mallet percussion and produce the haptic sense. Meanwhile, the visual feedback chime vibration was produced by mechanical simulation and the sound components musical scales were activated by the vibration jointly bring sensory experiences and virtual haptic senses due to psychological perception. Therefore, the haptic senses are escalated to the emotional identity level, and the process from physical senses to psychological identity is completed.

#### 3.3.3. Interactive Control

**Figure 5:**
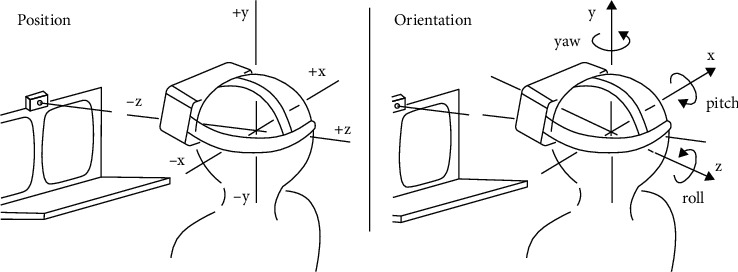
The mapping of the Unity3D coordinate system on position recognition.

*Position Recognition*. When users turn around or walk around wearing the headset, the renderer dynamically calculates the MVP composite matrix of each object through the vision-projection matrix at each frame and finally draws the vertices and image elements. Meanwhile, the 3D scene should be aware of the existence of the user head.  Figure 5 shows the mapping of the Unity3D coordinate system on Position recognition, and Table 1 describes the physical data contained in 6-DoF. Through the transmission of physical data, scene can obtain the sensor information of the headset, for example, position, direction, velocity, and acceleration. In this way, the camera, equipped with physical data and the ability to perceive physical objects, is no longer just an observer.

Gamepad events can be divided into the following three categories:

- Sensor event: the sensor physically tracks the gamepad
- Button event: interactive behavior generated by clicking a button
- Control unit event: generated by the input of thumbstick and touchpad

By interacting with the virtual scene through holding the gamepad, users can directly obtain the direction, position, velocity, and acceleration of the gamepad, thereby tracking the direction and position and mapping the physical gamepad to the 3D VR world. Moreover, more diversified physical behaviors can be conducted. For instance, the force on the object can be calculated by multiplying acceleration by mass. Therefore, the movements of the arms can be well simulated.

*Simulation of Reality Physical Engine*. The immersive virtual chime system simulates Newtonian mechanics through installing the PhysX physics engine developed by NVIDIA in unity and sets the sound effect of the gavel striking the chime by means of parameters, such as mass, gravity, friction, and speed, so as to realistically simulate physical effects, such as collision and gravity, making the virtual chime more real and vivid.

In physical simulation, the rigidbody component enables the object to move under the control of the physical system, and the rigid body accepts external force and torque force to make sure that the object moves as if it were in the real world. By setting the attribute parameters of the rigidbody component, the mass, drag, angular drag, and gravity of the object are set, and the interactive operation between the NVIDIA physical engine and the other objects is completed. The collides component triggers the collision simulation to prevent rigid bodies without colliders from passing through each other.

## 4. Experiments and Discussion

### 4.1. User Experience

User evaluation is an important part to evaluate this application. The field observation and questionnaire survey are carried out to acknowledge visitors' behaviors; the previous visualization research about visitors' behaviors in the museum is also a great help [27]. The evaluation process can be divided into two parts, adapting to the immersive device and experiencing the application and atmospheres of affects. Besides, museum experts also played an important role in the survey and gave us much helpful information about visitors' behavior patterns.

#### 4.1.1. User Evaluation Content

*Adapting to the Immersive Device*. Multisensory virtual chime music experience pavilion is a virtual chime music immersive experience environment built on the VR helmet system (HTC VIVE), jointly developed by HTC and Valve. The VR environment uses head-mounted displays and handle as the hit tool to hit the chimes for playing. Therefore, it is necessary to evaluate the comfortableness immersion of users of different ages in using virtual wearable devices, the wearing habits of users from different backgrounds, whether those without experience of using virtual devices will accept wearing such devices, and their abilities of learning to use new devices.

*Experiencing the Application*. For an immersive virtual instrument display system used for museum exhibition, it is necessary to evaluate the user's understanding of the system, the professional levels of virtual cultural relic instruments, and scientific nature of the system.

Virtual chime bells' interactive experience should be able to provide its necessary functions. First, the experience test scale to obtain the experiment data and comprehensively judge the experience of the instrument simulation of the virtual chimes. Second, evaluation explored the role of virtual reality in experiencing the performance of ancient musical instrument and verified the effectiveness of VR method in studying the sound characteristics and performance skills of ancient music instrument chime bells. Finally, the comparison test method is to compare the experience with existing chime music playing experiences and understand the pros and cons of the VR-based interactive design, so as to offer data support for functional improvement.

*Effects of the Psychophysics*. Human psychophysics is the quantitative measurement of perceptions. In essence, it is simply a more sophisticated version of what humans have done since time immemorial: noticed and reflected upon what humans can see, hear, and feel. As a virtual instrument relic, a chime has the following sensory feedbacks on three aspects.

Visual sense is the first channel to obtain the information from the virtual chime bells exhibition hall. Among all senses, visual sense provides the largest proportion of sensory experience. Visual information includes interactive environment information (such as the interactive interface design or the visual presentation of mechanical simulation during interactive process), virtual scene information (visual impact of the scene), and other elements.

As it is the virtual simulation of an ancient musical instrument, auditory experience is an important indicator for the virtual chime bells exhibition hall's sensory experience. Chime bells, with its unique tone, charm, and ability to produce two tones with each bell, bring unique stimulation to auditory sense, which is the core part of virtual chime bells' art experience.

*Chime Bells Are Played Using a Mallet*. The tactile feedback from this small tool after striking is an indispensable sense. In the experiment, the tactile sense from tactile feedback interface was measured. At the same time, the subject evaluated the effect of virtual tactile sense, which was generated by the sensory experience and psychological perception caused by visual and auditory senses during the interaction with chime bells.

The experience in artistic activities is often a comprehensive experience, where the immediate physiological experience of the environment generated from multiple surface sensory organs goes into a higher level to become psychological and emotional experience.

Winkler and Faller found that high-quality sound, together with visual elements, could suggest visually perceived quality [27]. Characteristics of musical instrument relics are considered; a multidimensional evaluation model based on perceived experience of chime bells instrument is proposed in this paper within the psychophysics. As it is impossible to obtain absolutely accurate evaluation formula, the new method got the following equation to calculate the multisensory cultural experience by using the equation proposed by Chalmers and Ferko [28] and add an experience element considering the cultural experience context:

(1) $$\begin{array}{c} P=\;\omega V\left(t\right)+\omega A\left(t\right)+\omega T\left(t\right)-\omega E\left(t\right), \end{array}$$

where *P* is the perception of the environment as a whole, which is assessed from four aspects, visual sense (*V*), auditory sense (*A*), tactile sense (*T*), and participant's experience (*E*), and *ω*  denotes the weight of each sense, and the weight of each index is processed with Kano model, ∑*ω*_*i*_=1.

The maximum threshold of each perceptual weight would not exceed the extent sensed in the real environment. According to signal detection theory, perception depends on knowledge and experience. The human consciousness comes from the primitive message of the external world and is conditioned by the past experience. For this reason, the new formula better corresponds with the psychophysics theory in the cognitive psychology. The relation between perception and prior knowledge is manifested in the interpretation of scientific data. The research holds the opinion that if participants are with the familiar bell culture, then the feeling of freshness lowers, and there will be fewer perception effects.

#### 4.1.2. Questionnaire Design

The evaluation adopted anonymous questionnaire form. Questionnaire survey as a research method collects data based on written questions. It reflects the investigation targets and level, and it relates to whether the collected data are real and comprehensive and whether the results of the study are scientific and effective.

The design of questionnaire is the most critical and important core link in the process of questionnaire survey because it directly affects the accuracy of the survey results and the usefulness of the entire study. Specific to a virtual chime interactive system for multisensory experience, a cognition-environment-emotion assessment system is proposed to form an assessment scale for cultural relics of musical instruments.

In a human-computer interaction system, behavior is influenced by user characteristics, namely, the internal cognitive attributes of the user, and the external environment. Internal cognitions include user's knowledge background, cultural emotion, and others. The external environment includes the scene and media. These internal and external uncertainties exert influence on the user experience. Thus, the questionnaire was organized into two parts. In the first part, indicators refer to the interaction design theory of Lanir et al. [29], which is developed on the basis “five elements of interaction design: people, actions, tools or media, purpose, and scene.” The second part collected respondents' demographic information including gender, age, educational level, occupation, and visit times. Related indicators were then developed.

Cognitive science reveals the psychological cognitive activities of the user, so as to evaluate user's cognition and behavior. Professor Glovanna in the field of cognitive neuroscience believes that cognitive models do not directly seek its physiological support, but they should not contradict neurophysiological findings [30]. A cognitive psychologist Rupert believes that cognitive tasks are closely related to the environment, and cognitive tasks are not always done in the brain. If cognitive tasks can use relevant behaviors in the environment instead of studying complex neural mechanisms, complexity and difficulty of this cognitive task will be reduced. The “cognition-environment-emotion” evaluation model proposed in this paper is also a cognitive, which abandons the complex neural mechanism. The research is focused on operability. “Cognition” means the interactive object faced by the user, the interaction process, sensory feedback, and brain cognition available. The physiological element in art experience can be divided into visual, auditory, tactile, and other senses based on sensory channels. The physiological feelings can often be reflected in psychological feelings, that is, emotion. The “emotion” mentioned in this paper is the user's behavior with artistic and cultural awareness, including user's pleasure experience and musical emotion created when playing the target virtual instrument. Psychological experience is the reflection of the cognition through the environment. The key point is to introduce a dimension of environment to replace the complex neural mechanism. It is believed in this paper that the immersive interaction process of musical instrument relics belongs to the mode of “cognition-environment-emotion”; namely, immersive environment (virtual musical instrument simulation system) serves as a hub connecting user's cognition and emotion. The measurement indicators representation is designed according to the mode as shown in Table 2.

*Cognition Scale Indicators*. In the cognition scale, “role” means the subject of the cognitive task, and it represents the target users group. Research on this aspect includes work like user characteristic analysis. Due to the artistic professionalism of instrumental music relics, it is emphasized in this paper that user characteristic is one of the key factors affecting the user experience rating of the interaction system. “Behavior” means research on behaviors, such as user behavioral habits. It is an important means to get tasks feedback from users' products usability testing and evaluation. The perceptual feedback of the interaction process is the foundation of the system, which involves the user's understanding of the object and the special emotional elements of art and culture. All task designs must comply with requirements on this dimension.

*Environment Scale Indicators*. The environmental module contains three index dimensions. “Scene” is an important part of environment, and it includes all nonfunctionality. However, it is a media, which directly contacts the user and affects the user media to some extent. “Task” is a dimension expressing the user's goal and showing the user's intent, and it is the direct purpose for establishing the interactive system of virtual musical instrument relics, and the users may achieve their goals by performing tasks. “Technology” is the guarantee that the system is able to interact with users.

*Emotion Scale Indicators*. Emotion is the ultimate expression of the experience system, including the comprehensive physiological and the psychological perception generated by users' experience of virtual musical instrument relics. Emotional expression can be divided into three categories: pleasure of experience mode, music emotion, and cultural emotion. So, evaluation assessed the expression emotion in aspects including experience's pleasure, music emotion, and cultural identity. In addition, evaluation found out the enhancing effect of virtual tactile sense brought by the visual and auditory experience and psychological perception during human-computer interaction on tactile sense feedback.

*Pleasure*. The pleasure of experience mode is the key factor that determines whether the users are willing to experience virtual relic musical instrument. The pleasure includes the attraction of immersive display of cultural relics, satisfaction for experiencing virtual musical instrument relics, and user's expectation for the same type of works after using the virtual instrument cultural relics display system.

**Figure 6:**
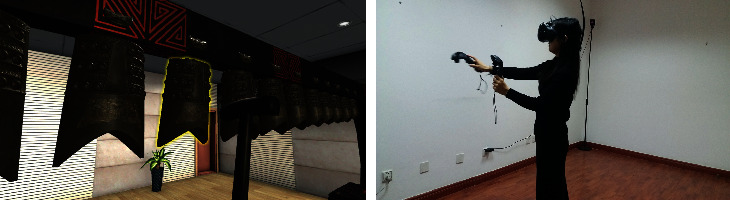
Examples of user test screenshots from virtual chime bells.

*Music Emotion*. Music emotion refers to the user's knowledge and understanding of instrumental music. As the subject of ritual and music culture unique to China, chime bells are an important testimony to the developmental history of ancient Chinese music, and their form and acoustics features reflect aesthetic consciousness and performance modes of China in different stages. Only when the user has certain instrumental music cognitive foundation and appreciation ability can user's corresponding aesthetic emotion be formed. Otherwise, instrumental music will be regarded as gratuitous noise. In order to enhance the emotional experience of music, multisensory chime bells playing experience system designs of two different modes, free experience mode and professional learning mode, to meet different needs of visitors and ancient instrument learners. In the learning mode, the bells are highlighted according to the choice of music step by step (Figure 6).

*Culture Influences*. Cultural emotion mainly refers to user's attitude and understanding of cultural background of cultural relics and recognition with the historical and cultural atmosphere related to cultural relics and the like.

As the ceremonial instrument in history, chime bells are played every time when there are activities, such as expedition, imperial visit, or sacrifice. There are detailed requirements for each performance. The cultural significance brought by chime bells to the participants has surpassed its musical instrument performance significance and contains rich cultural functions. As a ritual instrument, chime bells contain abundant thoughts about ritual and music culture and reflect aesthetic consciousness and spiritual pursuit smack of rites and music of that era. Cultural identity could be aroused when experiencing cultural and artistic content with national characteristics.

These indicators constitute the whole experience evaluation system.

### 4.2. Measurement Development

55 participants who have normal sensory functions are invited to join in the evaluation process. It was ensured that participants were provided with sufficient time to complete the test/questionnaire without any external interference. Personal information of 55 participants may influence the evaluation result, and their information is shown in Table 3. It can be seen from Table 3 that participants can be divided into several groups according to different classification standards, like gender, the highest academic degree, the age degree, immersive device using experience, and so on. The questionnaire result can also show these standards' influences to the evaluation result.

All the conditions of the participants were in line with the objective of this experiment. Participants were asked to follow the following steps:

1. To explore the rite and music culture and instrumental features of chime bells according to the guide interface
2. To complete the user experience and evaluate it using a 5-point Likert scale (1 = strongly disagree, 2 = disagree, 3 = sort of agree, 4 = agree, and 5 = strongly agree)

### 4.3. Results

**Figure 7:**
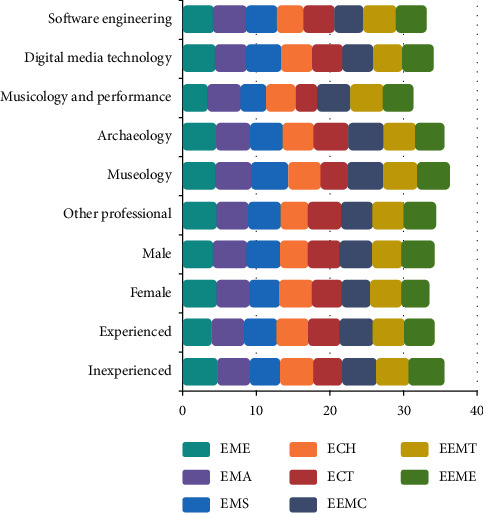
Assessment results of the emotion scale.

The assessment results of virtual bell performance experience are shown in Figure 7. It was found that participants majoring in music tended to complete simulation rapidly after acquiring detailed system operations, while participants majoring in other subjects who were not familiar with chime bells paid more attention to the system content and were more curious. This result indicates that participants who were more familiar with the culture consider the virtual environment less fresh and have less experience. In order to avoid evaluator effect, the scores of users with both musical cultural and professional background were analyzed separately. The evaluation results showed that the testers with musical cultural background and familiar with virtual environment gave lower scores for the experience of the system because the two had overlapping effects on the experience of the testers, causing less freshness. It is consistent with the effect of cultural background on overall experience as assumed in (1). It is worth noting that compared with participants majoring in other subjects, the participants majoring in music were more concerned about the instrumental feature of the virtual bronze chime bells (such as “one bell with two tones”). Overall, participants had good experience with the virtual chime bells exhibition hall. Most of them were satisfied psychologically. They felt strong cultural identity during virtual chime bells experience; the main score of cultural identity reached 4.65 points, indicating that virtual cultural products can produce educational and guiding effect similar to those of real cultural products. In addition, the score given with respect to virtual exhibition hall's visual effects, auditory effects, and interactive immersion was higher than 4.8 points. The scene score of the four indicators about instrument function's simulation reached 4.27 points.

Besides, gender and immersive device using experience can also influence the cognition result, the average score of male participants is larger than the score of female participants, and the average score of participants who have contacted immersive devices is lower than the score of those have not contacted immersive devices. It can be inferred from the text comments in the questionnaire that female participants have less interest in immersive devices than male participants, and participants who have not contacted immersive devices are more interested in those who have contacted immersive devices before, so in the opinion of these participants more interested in immersive devices, each aspect in the application is more excellent, and it may lead to score difference to a certain degree.

The study also explored the psychological implications of virtual tactile sense brought by the visual and auditory experience. To explore the influence of tactile feedback of virtual reality devices for participants' haptic perception, we set the four different versions of the experience, what was asked to assess the effect of visual factor (mechanical simulation), auditory factor (striking sound effect), tactile feedback, and multisensory experience on the tactile sense of the virtual chime bells' performance, respectively. In the evaluation, two evaluation parameters were set, namely, the impact on user experience (0 = low, 1 = medium, and 2 = high) and the impact on virtual playing music (0 = low, 1 = medium, and 2 = high). The psychological perception scores of user experience (with the value ranging from 0 to 10) were obtained by summing the two scores of each user. According to the Lewis magic number 5 theory [31], most or 80% of the usability problems can be found out by a test of 5 participants. In the study, 10 participants were tested, and the total score of single evaluation was 100.  Table 4 shows the average score for tactile experience without striking sound effect was 55.00, indicating a significant effect of striking sound effect on user's experience with virtual chime bells. The average score for tactile experience without mechanical simulation was 71.00, indicating that visual factors could bring deeper visual tactile impact and psychological identity. As virtual chime bells simulated the tactile sense of mallet striking bells, the effect of tactile feedback on the overall experience was relatively small. The average score for tactile experience without tactile feedback was 79.13. The study shows that virtual haptic and virtual feedback of virtual reality devices have psychological implications for participants' haptic rendering. When the virtual tactile perception together with tactile feedback could improve the ability of haptic rendering, this results in verified equation (1).

**Figure 8:**
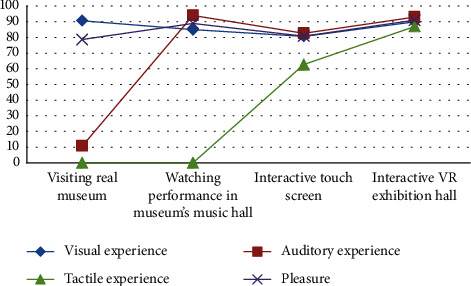
Results of the experience contrast for different methods.

Immersive technologies provide cultural heritage scholars and musicologists with a novel research way on 3D digital equivalents of ancient instrument. Based on the comparative human perception in chime bells' several different performance modes, the assessment scores show that watching real chime bells exhibits brought the highest satisfaction in terms of visual experience and provided low pleasure level due to a lack of auditory and tactile experience. Multitouch technology for chime bells' performance had limitations on cognitive experience, having a lower pleasure score than that of watching actors' performance. Virtual chime bells exhibition hall obtained a higher score for tactile experience. Though it had a lower score for auditory experience than that of actor performance, its score for pleasure level was the highest. The average experience with different methods is shown in Figure 8. The pleasure scores showed that through experiencing chime bells playing in a virtual exhibition room, experiencers can feel more satisfied and delighted than other ways to experience chime bells ritual and music culture (such as watching physical exhibits and musical performance, touch-screen interaction, and holographic laser projection interaction). For the experience of chime bells' rite and music culture, the realness of performance and completeness of sensory feedback had a significant impact.

## 5. Conclusion

Human perceives the environment through visual, auditory, tactile, and other sensory organs. The integrated experience of them can affect our psychological perception of the environment. Multisensory perception is richer than the sum of each sensory experience. Compared with traditional virtual chime bells' performance, immersive virtual reality can bring high-fidelity and multisensory experience with more natural sensory stimulation.

This research showed that sense organs will interact with each other, and the cooperation between high-quality sound and visual elements can increase tactile perceived quality, as high-level perception and interactive feedback are closely related to satisfaction. Virtual chime bells exhibition hall allows experiencers to feel the connotations of ritual and music culture of ancient chime bells and boost their interest and pleasure.

Virtual chime bells exhibition can bring this large cultural relic back to the public. It not only solves the limitations of large cultural relics in time and space dimensions but also improves digital research experience of ancient Chinese music and art. This attempt to develop new digital experiencing method in virtual environment has promoted the development of chime bells as a kind of digital entertainment.

Virtual chime bells can also be deployed in colleges and universities that make a specialty of music theory about chime bells and chime bells-playing skills. For musicology researchers, it is appealing to further study and research chime bells culture with this virtual simulation system as a tool.

## Figures and Tables

**Table 1:** Description of the physical data of 6-DoF (degree of freedom).

Attribute	Description
Position	Such event returns the headset and gamepad position
Orientation	Such event returns the headset and gamepad orientation
Angular acceleration	Such event returns the angular acceleration of the *x-*, *y-,* and *z-*axes
Angular velocity	Such event returns the angular velocity of the *x-*, *y-,* and *z*-axes
Linear acceleration	Such event returns the linear acceleration of the *x-*, *y-,* and *z*-axes
Linear velocity	Such event returns the linear velocity of the *x-*, *y-*, and *z*-axes

**Table 2:** Experience assessment scale.

Unit	Dimensions	Factors	Evaluation outline
Cognition	Role	Experience CRE	Does the user have experience of playing percussion instruments or music learning background? (Skills and knowledge background.)
	Behavior	Validity CBV	Is the user's operation valid? (Give a command to the system.)
		Natural CBN	Is the operation consistent with the user's behavior habit? (Is the percussion natural?)
	Perceptions	Visual sense CPV	Is the visual effect of virtual chime bells exhibition hall almost real?
		Auditory sense CPA	Are the sound of virtual chime bells accurate?
		Tactile feedback CPT	Is there any appreciable tactile feedback in the process of percussing the virtual chimes?
		Correctness CPC	Are properties of the instrument shown correctly (such as two tones of a chime)?

Environment	Scene	Verisimilitude ESV	Is it almost real (vivid model, physics simulation, and real beating behavior)?
		Medium ESM	Are the interface and tools clear?
		Guidance ESG	Is the user correctly guided? (The user will not be confused.)
		Immersive ESI	Is the scene naturally immersed?
	Task	Expectation ETE	Is it to the experience expectation of the user (system able to express the instrumental experience)?
		Function ETF	Does the virtual instrument have the consistent performance function as the real instrument?
		Rationalization ETR	Does it comply with the cognition of target instrument by neurophysiology?
	Technology	Completion ETC	Can the system framework be fully implemented?
		Stability ETS	Is the interaction process of the virtual exhibition system stable?

Emotion	Music emotion	Expertise EME	Does it produce the same scale and timbre as the real instrument?
		Aesthetics EMA	Is it possible to play a piece of music in its entirety?
		Subservience EMS	Does the auxiliary performance function provide effective musical instrumental aesthetic emotion?
	Cultural emotion	History sensibility ECH	Does the user feel the cultural connotation of ancient China's rites and music?
		Transmissibility ECT	Does the VR-chime bells help spread and promote traditional cultures?
	Pleasure	Charm EEMC	Experiencing chime bells performance using this method is attracting to tourist.
		Delight degree EEMT	User felt comfortable and delighted when experiencing chime bells performance.
		Expectation EEME	Tourist wanted to experience similar cultural products when experiencing VR-chime bells performance.

**Table 3:** Personal information of participants in the evaluation process.

Classification standard	Option	Frequency	Percentage
Gender	Male	35	63.64
	Female	20	36.36

The highest academic degree	Junior school	8	14.55
	High school	6	10.91
	Bachelor	24	43.64
	Master	12	21.82
	Doctor	5	9.08

The age degree	11–20	9	16.36
	21–30	28	50.90
	31–40	14	25.45
	41–50	4	7.27

Professional background	Software engineering	15	27.27
	Digital media technology	7	12.73
	Musicology and performance	20	36.36
	Archeology	3	5.45
	Museology	6	10.91
	Other	4	7.28

Have contacted immersive device before or not	Yes	8	14.55
	No	47	85.45

Interested in immersive device applied in museum visiting	Yes	53	96.36
	No	2	3.64

**Table 4:** Assessment of virtual tactile sense's effect on tactile experience.

Type	Assessed experience	Mean
Virtual tactile perception	Without striking sound effect	55.00
	Without mechanical simulation	71.00

Tactile experience	Without tactile feedback	79.13
Whole	Multisensory experience	92.67

## Data Availability

The raw data supporting the conclusions of this article will be made available by the corresponding author, without undue reservation.
